# HOXB8 enhances the proliferation and metastasis of colorectal cancer cells by promoting EMT via STAT3 activation

**DOI:** 10.1186/s12935-018-0717-6

**Published:** 2019-01-03

**Authors:** Tingting Wang, Feiyan Lin, Xuecheng Sun, Lei Jiang, Ruibo Mao, Shenyue Zhou, Wenjing Shang, Ruichun Bi, Fengying Lu, Shaotang Li

**Affiliations:** 1 Department of Gastroenterology, The First Affiliated Hospital of Wenzhou Medical University, Wenzhou, China; 2 Central Laboratory, The First Affiliated Hospital of Wenzhou Medical University, Wenzhou, China; 3 Department of Colorectal Surgery, The First Affiliated Hospital of Wenzhou Medical University, Wenzhou, 325000 Zhejiang China

**Keywords:** Colorectal cancer, Epithelial–mesenchymal transition, HOXB8, Signal transducer and activator of transcription 3

## Abstract

**Background:**

Previous studies have demonstrated that the expression of homeobox8 (HOXB8) is higher in colorectal cancer (CRC) tissues than in normal tissues; however, the precise role of HOXB8 in human CRC cells remains to be elucidated.

**Methods:**

We generated lentiviral constructs to overexpress and silence HOXB8 in CRC cell lines, and examined their biological functions through MTT, wound healing, colony and transwell, expression of signal transducer and activator of transcription 3 (STAT3) and epithelial–mesenchymal transition (EMT) related factors through western-blot.

**Results:**

HOXB8 knockdown inhibited cellular proliferation and invasion in vitro as well as carcinogenesis and metastasis in vivo. HOXB8 also induced EMT, which is characterized by the down-regulation of E-cadherin and the up-regulation of Vimentin, N-cadherin, Twist, Zeb1 and Zeb2. Moreover, HOXB8 activated STAT3, which is known to play an oncogenic role in diverse human malignancies.

**Conclusions:**

Our results indicate that HOXB8 may be an independent prognostic factor in CRC. Therefore, deserved a deeper research.

**Electronic supplementary material:**

The online version of this article (10.1186/s12935-018-0717-6) contains supplementary material, which is available to authorized users.

## Background

Colorectal cancer, which is tightly linked to genetic alterations and lifestyle, ranks as the fourth leading cause of mortality among common cancers. The incidence rate of CRC is higher in developed countries than in developing countries [1–3]. Most patients with CRC are usually diagnosed at an advanced metastatic stage and may not be cured by surgery alone. Therefore, it is urgent to identify efficient biomarkers as well as potential mechanisms for the early diagnosis and targeted therapy.

The Hox gene family encodes proteins that act as transcription factors during embryonic development. This family of genes was initially found to play an important role in embryonic development in *Drosophila melanogaster* [4, 5]. Hox genes have been demonstrated to be associated with the development of various gastrointestinal cancers, such as CRC, hepatocellular carcinoma and gastric cancer [6, 7]. In vitro studies by Vider et al. have demonstrated that HOXB8, a member of the Hox family, is up-regulated in CRC cell lines. Moreover, the up-regulated expression of HOXB8 has been observed in all stages of CRC (including pre-cancerous polyps) [8, 9].

In this study, by constructing a HOXB8 lentiviral expression vector, we established cell lines with stable overexpression and knockdown of HOXB8. We found that the overexpression of HOXB8 promotes the proliferation, migration and invasion of CRC cells, while the silencing of HOXB8 leads to the opposite effects. We investigated the relationship between HOXB8 expression and the occurrence or development of CRC, thereby laying the foundation for further studies on targeted therapies for CRC using HOXB8 and its associated regulatory mechanisms.

## Methods

### Cell culture

Human-derived colorectal cancer cell lines HCT-116, SW480, RKO and DLD1, normal colonic epithelial cell line NCM460, and human embryonic kidney 293T were purchased from the Chinese Academy of Sciences, Shanghai, China. HCT-116 was maintained in McCoy’s 5a Medium supplemented with 10% fetal bovine serum and 100 U/ml penicillin/streptomycin (Invitrogen, Carlsbad, CA. USA). RKO cells were maintained in DMEM with the same supplements, SW480, DLD1 and NCM460 cells were maintained in RPMI 1640 medium with the same supplements. All cells were maintained in humidified 37 °C incubator with 5% CO_2_. When cell confluency reached approximately 80–100%, we harvested and passaged the cells with 0.25% trypsin for the following experiments.

### Chemicals and antibodies

HOXB8 antibody was purchased from biorbyt (San Francisco, California, USA), Zeb2 antibody was purchased from proteintech (Chicago, IIlinois, USA), CD31, Twist and N-cadherin antibody were purchased from abcam (Cambridge, MA, USA), phospho-STAT3(p-STAT3), STAT3, E-cadherin, Vimentin, Ki67 and Zeb1 antibodies were purchased from Cell Signaling Technology (Danvers, MA, USA), p-STAT3 inhibitor (S3I-201) was purchased from Slleckchem (Houston, TX, USA), TRIzol, and SYBGreen fluorescent quantitation PCR reagent were purchased from Invitrogen (Carlsbad, CA, USA), Superscript First-Strand Synthesis Kit were purchased from Promega (Madison, Wisconsin, USA). 1% crystal violet staining solution was purchased from Sangon Biotech (Shanghai, China). Bouin’s solution was purchased from Solarbio (Beijing, China), the enhanced chemiluminescent Thermo Scientific Super-Signal West Femto were purchased from Thermo Fisher Scientific (Waltham, MA USA). Color one-step rapid mycoplasma detection kit was bought from Yeasen (Shanghai, China).

### Lentiviral vector construction and transduction

Lentiviral vectors stored in our laboratory. Total RNA of RKO was extracted using TRIzol (Invitrogen). We measured its 260 nm/280 nm absorbance value on Enzyme standard instrument, calculated the concentration and selected 260 nm/280 nm absorbance value between 1.8 and 2.0 as the template to get HOXB8 full-length cDNA by RT-PCR using Superscript First-Strand Synthesis Kit (Promega) with specific designed primers (HOXB8-F-*Eco*RI: 5′-CGA ATT CGC CAC CAT GAG CTC TTA TTT CG TCA AC-3′, HOXB-R-SAC II: 5′-CCG CGG CTA CTT CTT GTC GCC CTT CTG-3′). The PCR products were purified and then inserted into the *Eco*RI/*Sac*II sites of lentiviral vector. The inserted fragment was confirmed by sequencing. We used Lentiviral vectors expressing EGFP as a control. Two short-hairpin RNA (shRNA) were designed targeting HOXB8:shHOXB8-1 (5′-GCTCTTATTTCGTCAACTCACTGTTCTCC-3′) and shHOXB8-2 (5′-GAGCTGGAGAAGGAGTTCCTATTTAATCC-3′). Briefly, Lenti-shRNA vector construction was done as follow: The DNA fragments containing CCAA were synthesized as the loop for shRNA, then we cloned the shRNA into human pBluescript SK (+) plasmid (pU6) with U6 promoter and inserted the U6-shRNA cassettes into an appropriate lentiviral vector. Similarly, we selected a control: Lentiviral vector carrying shRNA targeting firefly luciferase (shLuc: 5′-TGC GCT GCT GGT GCC AAC CCT ATT CT-3′). The transfer vector and the other three packaging vectors (pMD2.G, pMDL-G/P-RRE and pRSV-REV) were co-transfected into 293T cells to produce the viral particles. The concentration of each vectors was added as follow: pMD2.G 3.5 mg/10 cm, pMDL-G/P-RRE 6.5 mg/10 cm, pRSV-REV 3.5 mg/10 cm and Transfer Vector 12 mg/10 cm. After 48 h the supernatant was collected and subsequently purified using ultracentrifugation to get high-quality viral particles. The first day we seeded 5 * 10^4^ cells in 24-well plates, and the next transduced with lentivirus (multiplicity of infection, MOI = 5) supplemented with 8 μg/ml of polybrene (sigma-Aldrich Chemie, The Netherlands). Transfection efficiency was verified by western blot and Real-time Polymerase Chain Reaction.

### Real-time PCR

Total RNA was purified from cells using TRIzol (Invitrogen) according to the manufacturer’s instructions. Equal amounts of RNA (500 ng) were reverse-transcribed using a cDNA synthesis kit (Promega). Diluted cDNAs (1:5 final) were subjected to qPCR analysis using SYBR Green Supermix reagent (Invitrogen). The relative amount of HOXB8 expression was normalized to an endogenous housekeeping gene GAPDH. Primer sequences: HOXB8-F: 5′-TAA GCG GCG AAT CGA GGT AT-3′; HOXB8-R: 5′-TGT TTC TCC AGC TCC TCC TG-3′. GAPDH: 5′-CCA GCC GAG CCA CATCGC TC-3′ and 5′-ATG AGC CCC AGC CTT CTC CAT-3′.

### Western blot

Protein was extracted by using lysate. Conducted protein quantification with reference to BCA protein concentration assay kit. Took 30 μg of each sample for SDS-polyacrylamide gel electrophoresis and transferred to polyvinyl difluoride membranes (Millipore, Billerica, MA, USA). Blocked with 5% nonfat dry milk in tris-buffered saline containing 0.1% Tween-20 (TBST) for 1 h, 4 °C overnight incubation using primary antibodies, HOXB8 (1:1000), p-STAT3 (1:1000), STAT3 (1:1000), E-cadherin (1:1000), Vimentin (1:1000), N-cadherin (1:1000), Twist (1:1000), Zeb1 (1:1000), Zeb2 (1:1000) and internal-reference GAPDH (1:1000), used goat anti-rabbit or goat anti-mouse horseradish peroxidase (HRP)-conjugated secondary antibodies (Bio-world Technology, MN, USA) to hybridize at room temperature for 1 h, the membranes were visualized by ECL (Millipore, MA, USA) and finally conducted the imaging by using the gel imaging system.

### Mycoplasma detection

The cells (DLD1-Control, DLD1-GFP, DLD1-HOXB8, SW480-Control, SW480-shLUC, SW480-shHOXB8-1, SW480-shHOXB8-2, HCT116-Control, HCT116-shLUC, HCT116-shHOXB8-1, HCT116-shHOXB8-2) grew to 80%–90% and cultured for 3 days continually, got the medium to color one-step rapid mycoplasma detection kit (Yeasen) according to the manufacturer’s instructions. If the cancer cells were infected by mycoplasma, the color will turn to navy blue as the positive control, otherwise, it will turn to blue purple as negative control. The result was showed in Additional file 1: Figure S1.

### MTT assay

DLD1-Control, DLD1-GFP, DLD1-HOXB8, SW480-Control, SW480-shLUC, SW480-shHOXB8-1, SW480-shHOXB8-2, HCT116-Control, HCT116-shLUC, HCT116-shHOXB8-1, HCT116-shHOXB8-2 were inoculated into 96-well plates (2000 cells per well), at different time points (days 1, 2, 3, 4 and 5), the culture medium was removed and replaced with culture medium containing 10 μl of sterile MTT dye (5 mg/ml). After incubation at 37 °C for 4 h, the MTT solution was removed, and 150 μl dimethyl sulfoxide (DMSO) was added to each well followed by measuring the absorbance at 570 nm on an enzyme immunoassay analyzer (Bio-Rad).

### Colony formation

DLD1-Control, DLD1-GFP, DLD1-HOXB8, SW480-Control, SW480-shLUC, SW480-shHOXB8-1, SW480-shHOXB8-2, HCT116-Control, HCT116-shLUC, HCT116-shHOXB8-1, HCT116-shHOXB8-2 were inoculated into 6-well plates (500 cells per well) respectively, cultured with CO_2_ (5%) at 37 °C for 2 weeks, washed the plates using 1 × PBS three times and fixed them with 4% paraformaldehyde for 30 min, finally stained the cells using 0.1% of crystal violet (Sangon Biotech), and observed the changes in number and size of the cell colony.

### Wound healing assay

DLD1-Control, DLD1-GFP, DLD1-HOXB8, SW480-Control, SW480-shLUC, SW480-shHOXB8-1, SW480-shHOXB8-2, HCT116-Control, HCT116-shLUC, HCT116-shHOXB8-1, HCT116-shHOXB8-2 were planted on 6-well plates and cultured as confluent monolayer. The cells were carefully scraped using a 20 μl pipette tip and debris was removed by washing with 1 × PBS. Cell migration was evaluated at 48 h, and observed the changes in size using an inverted microscope.

### Transwell assays

DLD1-Control, DLD1-GFP, DLD1-HOXB8, SW480-Control, SW480-shLUC, SW480-shHOXB8-1, SW480-shHOXB8-2, HCT116-Control, HCT116-shLUC, HCT116-shHOXB8-1, HCT116-shHOXB8-2 were seeded into upper chamber (Corning, Cambridge, MA, USA) with serum-free medium (2 * 10^5^ cells per well), the inserts were coated with 10 μl of 1 mg/ml Matrigel matrix l (BD Biosciences, Bedford, MD, USA) according to the manufacturer’s recommendations, in the lower chamber there was 800 μl of complete medium. Then chambers were incubated in the humidified 37 °C incubator (5% CO_2_) for 24 h, removed the non-migratory cells in the upper chamber and fixed them with 4% paraformaldehyde for 30 min, after all, stained the migrated cells below with 0.1% of crystal violet (Sangon Biotech) for 20 min. The migrated cells were counted under an inverted microscope. Migration assays were similar to Matrigel invasion assay except that the transwell insert was not coated with Matrigel.

### Mice model establishment

6-week-old female nude mice were obtained from the Shanghai Slac Laboratory Animal Co. Ltd and kept in Wenzhou medical university animal lab for 1-week accordance with the requirements of the German Animal Welfare Act (reference number: G11.559). HCT116-shHOXB8-1 and its correspond control cells (2 * 10^6^/0.2 ml) were injected subcutaneously into the right flank of 7 mice respectively. Tumor size was measured every 3 days to calculate the respective tumor volume using the following formula: volume = (width^2^ * length)/2. Mice were sacrificed at 4 weeks. To evaluate long-distance metastasis, HCT116-shHOXB8-1 and its correspond control cells (3 * 10^6^/0.2 ml) were injected into 7 nude mice by way of tail vein to imitate long-distance metastasis. Mice were sacrificed at 7 weeks, and the metastases were fixed by Bouin’s solution (Solarbio) and confirmed by H&E staining.

### Immunohistochemistry analysis

Immunohistochemistry was performed using Ki-67, CD31 and HOXB8 antibodies. Tissue sections were deparaffinized in xylene and rehydrated with ethanol, then blocked with 10% normal goat serum in 1 × PBS (pH 7.5) followed with incubation with primary antibody overnight at 4 °C. Tissue sections were stained with biotinylated secondary antibody (Vector lab, Burlingame, CA) for 0.5 h at 37 °C. The peroxidase reaction was developed with diaminobenzidine (DAB kit, Vector lab) and the slides were counterstained with hematoxylin. Representative images were captured with a Leica DM4000B microscope (Jena, Germany).

### Statistical processing methods

SPSS17.0 (IBM, Armonk, NY, USA) and Prism 5.0 software (GraphPad Software, San Diego, CA, USA) were used in the experiment. Each set of experimental data was represented as “mean ± SD”, *s*tudent’s *t* tests were applied to analyze the statistical significance between groups. *P* < 0.05 was accepted for statistical significance.

## Results

### HOXB8 is overexpressed in CRC

**Fig. 1:**
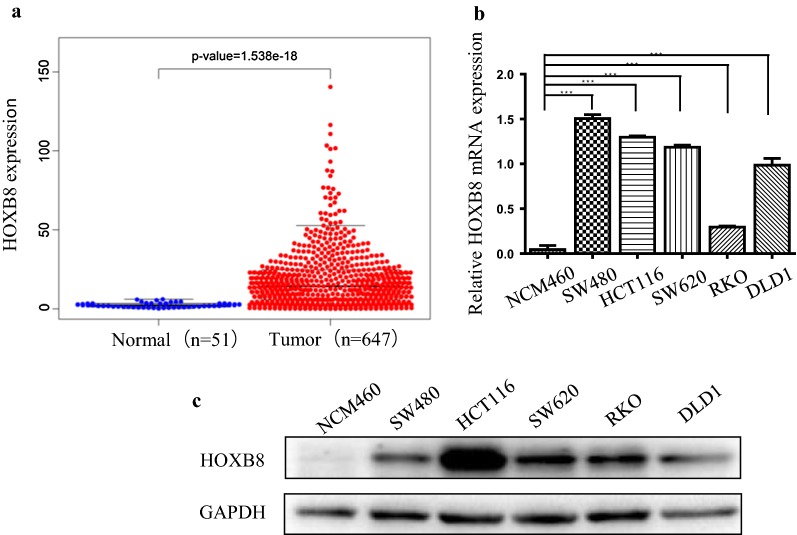
HOXB8 is high expression in CRC tissues and cells. **a** HOXB8 expression was significantly increased in CRC tumor tissues when compared with normal tissues from TCGA. **b**, **c** HOXB8 expression in colorectal cancer cell lines was analyzed by qRT-PCR (**b**) and Western blot (**c**), among them, SW480 and HCT116 showed highest expression of HOXB8 protein and mRNA, DLD1 showed median expression of HOXB8 mRNA and lowest expression of HOXB8 protein. ***P < 0.001

To test whether HOXB8 expression correlates with CRC, multiple microarray datasets of CRC from The Cancer Genome Atlas (TCGA) were analyzed, and we found that the HOXB8 expression level was significantly higher in CRC tissues than in normal tissues (Fig. 1a). Furthermore, to determine the expression level of HOXB8 in CRC cell lines, RT-PCT analysis was performed. Compared to a normal colorectal mucosa cell line, NCM460, HOXB8 mRNA expression was up-regulated in CRC cell lines such as SW480, HCT116, SW620, RKO and DLD-1 (Fig. 1b). In addition, the level of HOXB8 protein, evaluated by western blot, was also up-regulated in these CRC cell lines (Fig. 1c). Taken together, these results suggested that HOXB8 might be involved in CRC.

### Up-regulation of HOXB8 promotes the proliferation and invasion of CRC cell lines

**Fig. 2:**
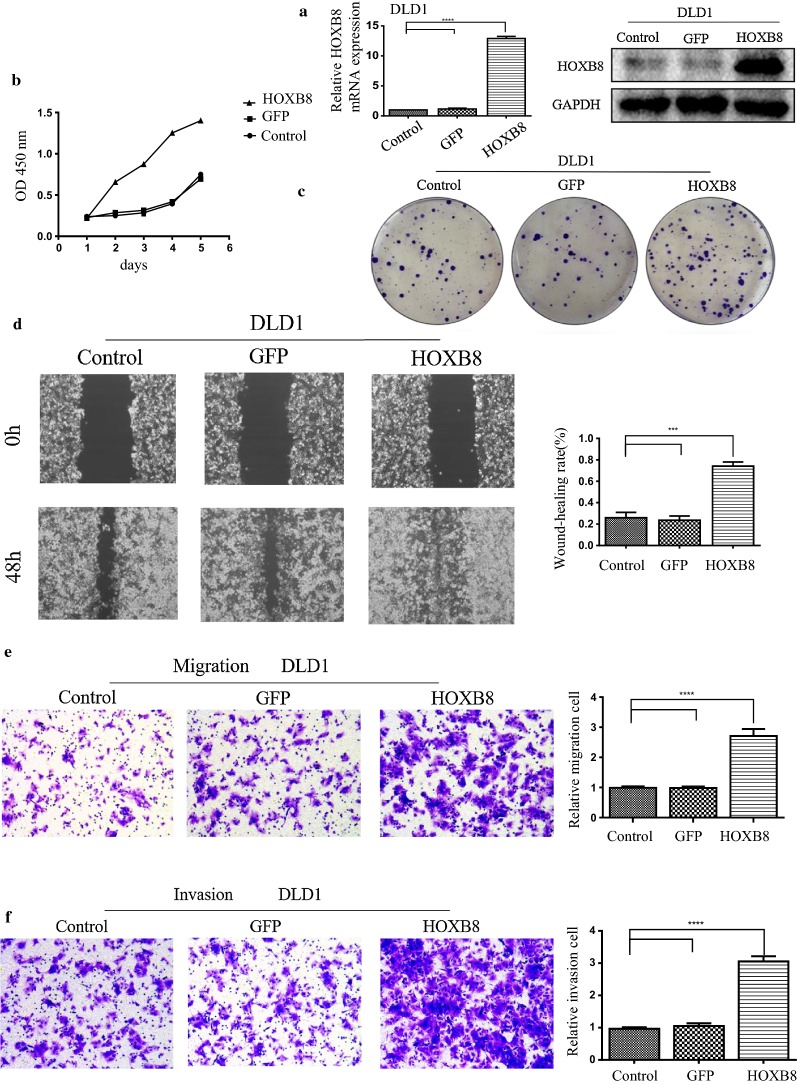
HOXB8 increases the proliferation and invasion capacity of CRC cells. **a** Stable overexpression of HOXB8 CRC cell lines were established in DLD1, and verified by qRT-PCR (left), Western blot (right). **b**, **c** CRC cells with high expression of HOXB8 possessed stronger proliferation ability in MTT (**b**) and in colony (**c**). **d**–**f** CRC cells with overexpression of HOXB8 possessed more migrated and invasion abilities in wound healing (**d**), transwell assay (**e**, **f**), Statistical result of each experiment is also shown on the right side respectively. ***P < 0.001, ****P < 0.0001

While successfully constructed the overexpression of HOXB8 in DLD1 cell (Fig. 2a), we performed MTT and colony formation assays to evaluate the effects of HOXB8 on the proliferation of CRC cells, and we also evaluate the effect of HOXB8 on the migration and invasion of CRC cells by performing wound healing and transwell assays. The results showed that overexpression of HOXB8 promoted cellular proliferation (Fig. 2b, c) and the migration and invasion of CRC cells (Fig. 2d–f). These results indicated that HOXB8 promotes the proliferation and invasion of CRC cells in vitro.

### Knockdown of HOXB8 inhibits the proliferation and invasion of CRC cell lines in vitro

**Fig. 3:**
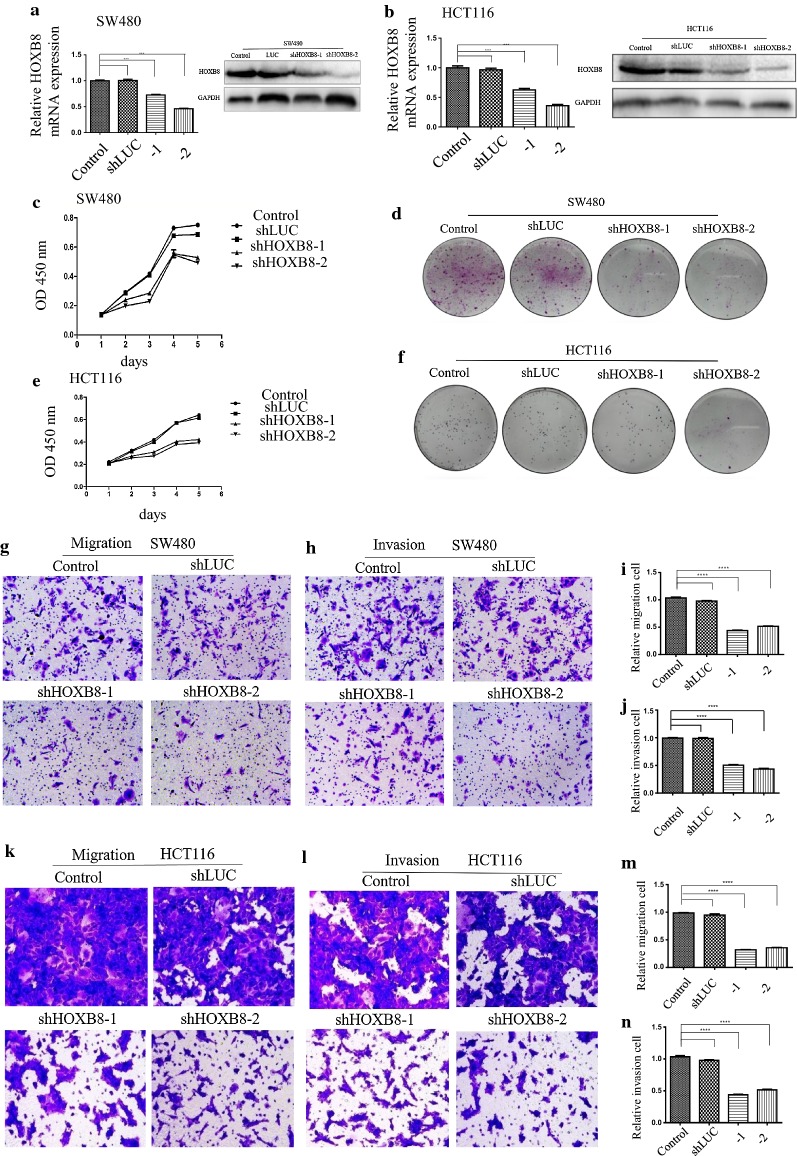
Silencing HOXB8 inhibits the proliferation and invasion capacity of CRC cells. **a**, **b** Table knockdown of HOXB8 CRC cell lines were established in SW480 and HCT116, and verified by qRT-PCR (left), Western blot (right). **c**–**f** The proliferation ability of CRC cells with low expression of HOXB8 possessed weaker proliferation ability in MTT (**c**, **e**) and in colony (**d**, **f**) both in SW480 and HCT116. **g**–**n** The migration and invasion abilities were tested in transwell assay in SW480 (**g**, **h**) and HCT116 (**k**, **l**), Statistical result of each experiment is also shown on the right side both in SW480 (**i**, **j**) and HCT116 (**m**, **n**) respectively. ***P < 0.001, ****P < 0.0001

To further verify the biological function of HOXB8 in CRC cell lines, HOXB8 was silenced in the cell lines HCT116-shHOXB8-1/-2 and SW480-shHOXB8-1/-2 (Fig. 3a, b). Knockdown of HOXB8 significantly inhibited the proliferation in both cell lines (Fig. 3c–f) and also their migration and invasion (Fig. 3g–n).

### Knockdown of HOXB8 inhibits tumorigenesis and metastasis in vivo

**Fig. 4:**
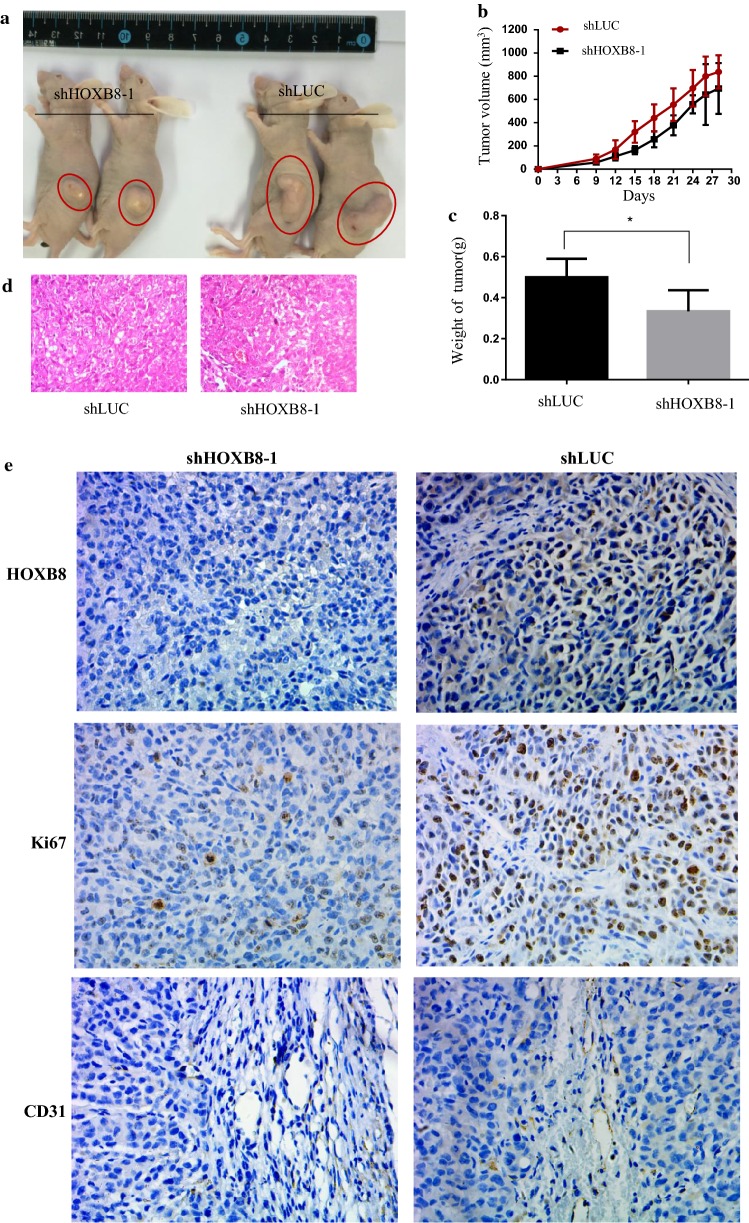
Silencing HOXB8 decreases tumorgenesis in vivo. **a** A representative image of xenograft tumor mice model was shown. **b** Tumor volume change tendency was drawed by software. **c** Weight of tumor volume was weighed and histogram was drawed by software. **d** Representative H&E results were carried out to prove the successfully establishment of nude model. **e** Representative IHC images of xenograft tumors mice model was shown in HOXB8, Ki67 and CD31 respectively. Scale bar = 50 μm. *P < 0.05

**Fig. 5:**
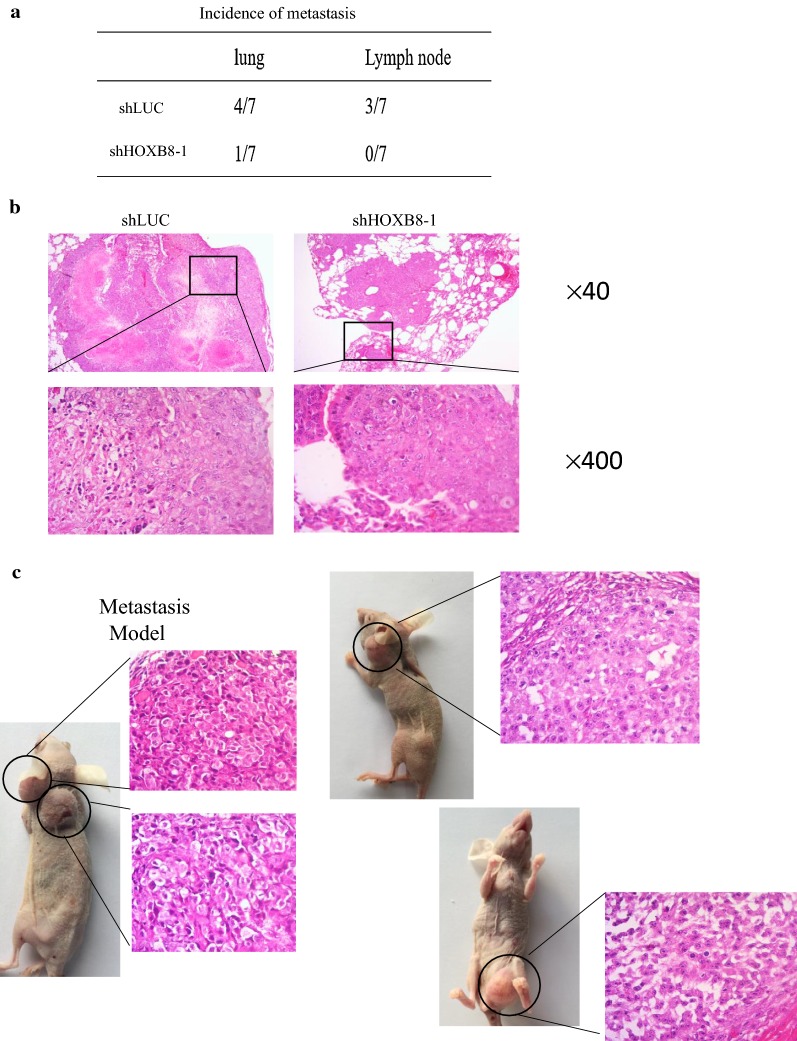
Silencing HOXB8 decreases metastasis in vivo. **a** Less number of mice with distant metastasis (lung, lymph node) were found in mice injected with HCT116-shHOXB8-1 cells. **b** Typical metastasis tumors of lung were proved by H&E (scale bar = 400 μm above and scale bar = 50 μm below). **c** All lymph node metastasis mice were shown and tumors were proved by H&E. Scale bar = 50 μm

Next, we aimed to explore the function of HOXB8 in tumor growth in vivo. HCT116-shHOXB8-1 and its control were subcutaneously injected into the right flanks of nude mice. Silencing of HOXB8 inhibited tumor growth (Fig. 4a–c), all these tumors were further verified by H&E staining (Fig. 4d). Immunohistochemical (IHC) analysis showed that the proportion of Ki-67-positive cells and CD31-stained vessels was lower in HOXB8-knockdown tumors than in control tumors (Fig. 4e). To further verify the function of HOXB8 in distant metastasis in vivo, HCT116-shHOXB8-1 and its control were injected into the tail veins of nude mice. Indeed, knockdown of HOXB8 decreased not only lung metastasis but also lymph node metastasis (Fig. 5a–c), these metastasis tumors were verified by H&E staining.

### HOXB8 induces EMT by activating STAT3

**Fig. 6:**
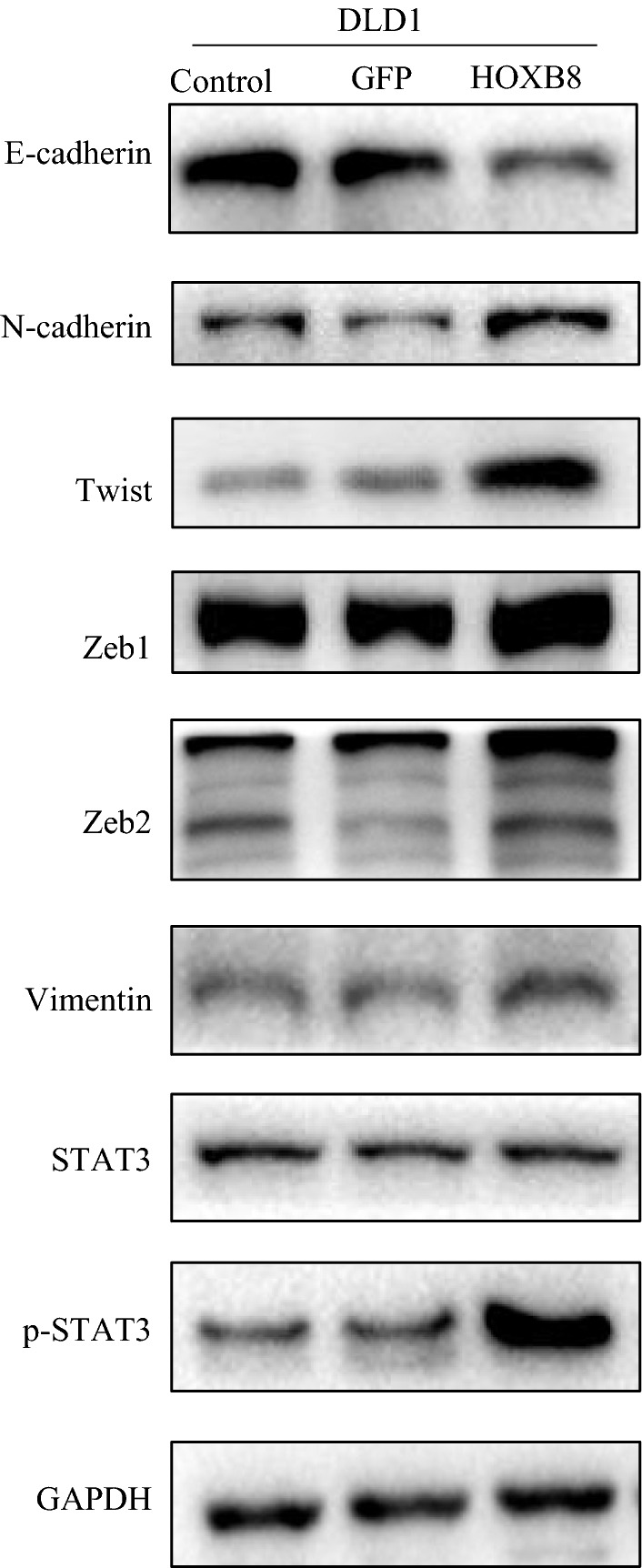
Proteins involved in EMT and STAT3 pathway were determined by western-blot analysis in DLD1 cell lines. HOXB8 overexpression decreased the expression of E-cadherin and increased the expression of Vimentin, N-cadherin, Twist, Zeb1 and Zeb2.p-STAT3 is high expression along with the high level of HOXB8 expression while STAT3 expression stays stable

**Fig. 7:**
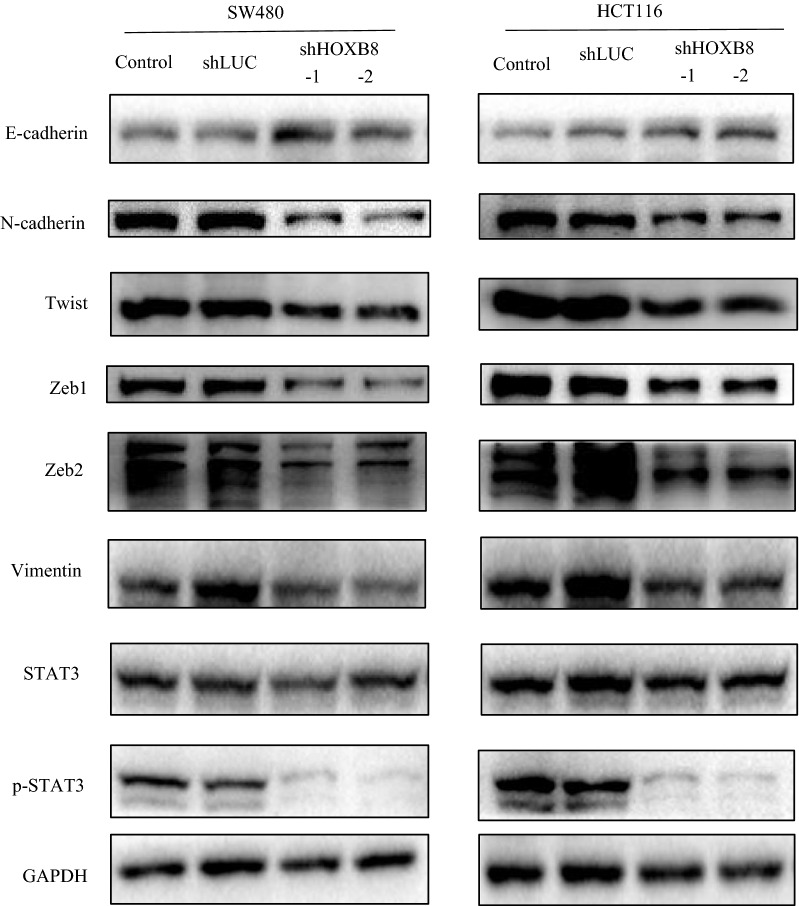
Proteins involved in EMT and STAT3 pathway were determined by western-blot analysis in both in SW480 and HCT116 cell lines. HOXB8 depletion increased the expression of E-cadherin and decreased the expression of Vimentin, N-cadherin, Twist, Zeb1 and Zeb2.p-STAT3 is low expression along with the low level of HOXB8 expression while STAT3 expression stays stable

**Fig. 8:**
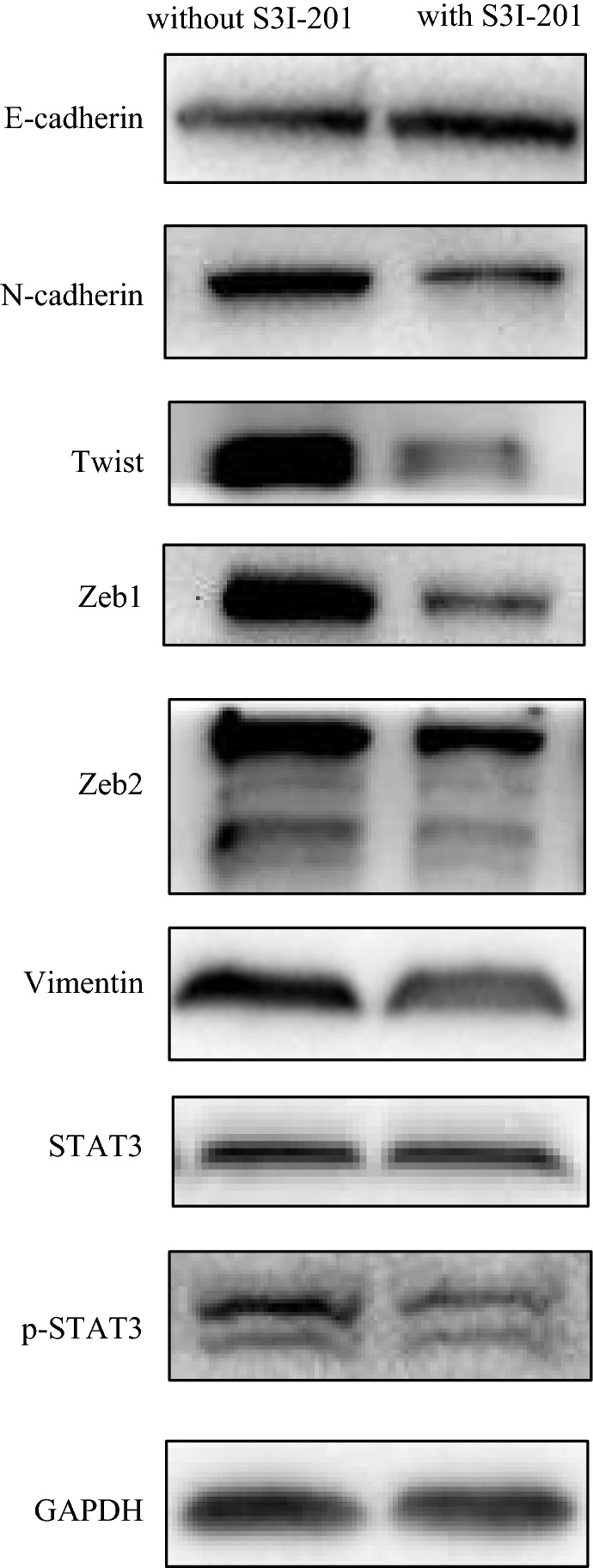
Proteins involved in EMT and STAT3 pathway were determined by western-blot analysis with the use of inhibitor. DLD1-HOXB8 treated with p-STAT3 inhibitor after 12 h, DLD1-GFP without any treatment stands for a control. P-STAT3 inhibitor increased the expression of E-cadherin, and decreased the expression of Vimentin, N-cadherin, Twist, Zeb1 and Zeb2

Based on the above results, HOXB8 markedly enhanced the motility of CRC cells especially the proliferation and invasion ability. Additionally, STAT3 is known to play an oncogenic role in many malignancies including CRC. In western blot, we found that HOXB8 overexpression decreased the expression of E-cadherin and increased the expression of Vimentin, N-cadherin, Twist, Zeb1 and Zeb2, and p-STAT3 levels were positive correlated with HOXB8 levels, despite stable expression of STAT3 (Figs. 6, 7). p-STAT3 inhibitor (S3I-201) was used to treat DLD1-HOXB8 for 12 h, the expression of p-STAT3 was reduced compared to untreated DLD1-GFP, and EMT was reversed (Fig. 8) what’s more, STAT3 inhibitor also affectted the mobility of HOXB8-overexpression cells (Additional file 2: Figure S2).

## Discussion

Hox proteins are important transcription factors in embryonic development and differentially expressed in adult tissues. Studies have shown that the Hox gene family is associated with various cancers [10–12] and all stages of cancer occurrence and development, especially in CRC [8, 9, 13]. However, the role of HOXB8, a member of the Hox gene family, is inconclusive thus far. In the experiment, we have successfully confirmed that HOXB8 is up regulated in CRC and promote the formation and metastasis of CRC cells both in vitro and in vivo. But how does HOXB8 make it’s functions? That is the point we need to further clarify.

Epithelial–mesenchymal transition is clearly known to be one of the factors in the migration and invasion of cancer cells [14]. Ding et al. have revealed that HOXB8 regulates the metastasis of gastric cancer cells by inducing EMT [7]. Our experiment also proved that HOXB8 markedly promote the proliferation and metastasis of CRC via EMT. Interestingly, in the distant metastasis model, we observed not only lung metastasis, which was considered as the common target organ for hematogenous metastasis, but also lymphatic metastasis. This observation indicated that HOXB8 might promote lymphatic metastasis, and lead to poor disease prognosis.

Signal transducer and activator of transcription 3 (STAT3), a key transcription factor, is overexpressed or constitutively activated by proinflammatory cytokines, growth factors and oncogenic proteins. The abnormal activation of STAT3 contributes to the progression of various human malignancies, including in CRC. Though it has not been proven to be a classic pathway interacting with EMT, STAT3 was shown to contribute to EMT through comprehensive alterations of transcription factors such as Zeb1 [15]. What’s more, Zhang et al. demonstrated that STAT3 may cooperate with Twist to mediate EMT and induce cancer invasion and metastasis [16]. In our present study, we are surprised to find that overexpression of HOXB8 significantly active the p-STAT3. Inhibition of p-STAT3 activity by S3I-201 inhibited EMT, as shown by increased levels of E-cadherin and reduced levels of N-cadherin, Twist, Vimentin, Zeb1, Zeb2. Therefore, we tentatively conclude that HOXB8 promotes EMT by activating the STAT3 pathway in CRC, revealing a potential connection between HOXB8 and EMT. However, further research is need to determine the specific mechanism that HOXB8 active STAT3 signaling in CRC.

Increasing evidences have demonstrated a close association of dysregulated STAT3 expression in human CRC chemotherapy responses [17]. Lu et al. established a prediction model based on seven genes (including HOXB8) that is highly accurate in predicting sensitivity to the chemotherapy regimen FOLFOX4 in CRC patients with liver metastases [18]. Our previous findings showing that after treated with 4 weeks standard FOLFOX4 chemotherapy, advanced metastatic CRC patients with HOXB8 higher expression results in stable disease or progressive disease, while lower expression group shows partial response [19]. Combined with our results in western blot, we hypothesis HOXB8 may activate STAT3 pathway, and be a useful and independent biomarker for predicting chemotherapy responses. Although chemotherapy is the main treatment modality for advanced metastatic CRC [20–22], approximately half of CRC patients do not respond to combination chemotherapy, and the majority of these patients eventually exhibit drug resistance; nevertheless, the reason for the occurrence of resistance has mostly been attributed to differences among individuals [23, 24], our findings may lead a further research in future chemotherapy resistance in CRC, and provide considerable insight into this disease.

## Conclusion

We hypothesize that HOXB8 activates STAT3 to regulate EMT and thus acts as an important transcription factor in the occurrence, development and progression of CRC, resulting in a poor prognosis. Thus, HOXB8 might serve as a therapeutic target in CRC and should be the focus of future studies into the biological and molecular mechanisms of CRC.

## Additional files

**Additional file 1: Figure S1.** The detection of mycoplasma.1 stands for positive control, the color is navy blue, 2 stands for DLD1, 3 stands for DLD1-GFP, 4 stands for DLD1-HOXB8, 5 stands for SW480, 6stands for SW480LUC, 7 stands for SW480shHOXB8-1, 8 stands for SW480shHOXB8-2, 9 stands for HCT116, 10 stands for HCT116LUC, 11 stands for HCT116-shHOXB8-1, 12 stands for HCT116shHOXB8-2,13 stands for negative control, the color is blue purple.

**Additional file 2: Figure S2.** p-STAT3 inhibitor (S3I-201) inhibit the motility of HOX8B-overexpressing cells. with S3I-201 treated for 12 hours, the cell number of DLD1-HOXB8 was decreased both in migration and invasion assays, compared to control.

## Abbreviations

CRC: colorectal cancer
EMT: epithelial–mesenchymal transition
HOXB8: homeobox8
MOI: multiplicity of infection
STAT3: signal transducer and activator of transcription 3
TCGA: Cancer Genome Atlas
IHC: immunohistochemical
