# Supplementary figures and images for: HOXB8 enhances the proliferation and metastasis of colorectal cancer cells by promoting EMT via STAT3 activation
Source: Cancer Cell Int. 2019 Jan 3;19:3. doi: 10.1186/s12935-018-0717-6 (PMC6317211; doi:10.1186/s12935-018-0717-6)

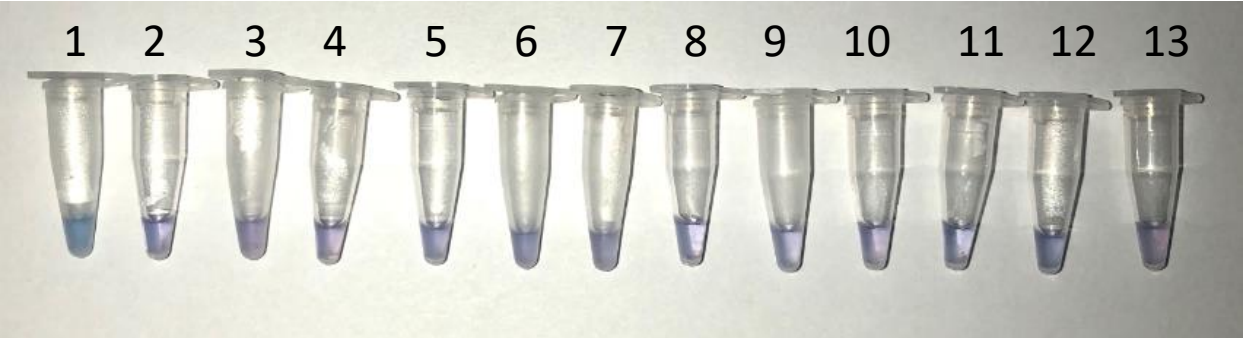

Supplement: Supplementary file 1 — Additional file 1: Figure S1. The detection of mycoplasma.1 stands for positive control, the color is navy blue, 2 stands for DLD1, 3 stands for DLD1-GFP, 4 stands for DLD1-HOXB8, 5 stands for SW480, 6stands for SW480LUC, 7 stands for SW480shHOXB8-1, 8 stands for SW480shHOXB8-2, 9 stands for HCT116, 10 stands for HCT116LUC, 11 stands for HCT116-shHOXB8-1, 12 stands for HCT116shHOXB8-2,13 stands for negative control, the color is blue purple. [file 12935_2018_717_MOESM1_ESM.pdf]

without S3I-201

with S3I-201

Migration

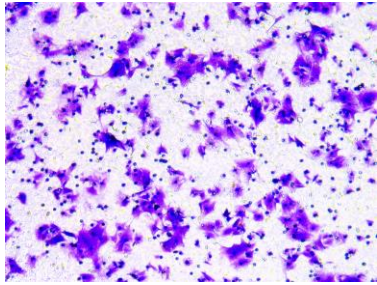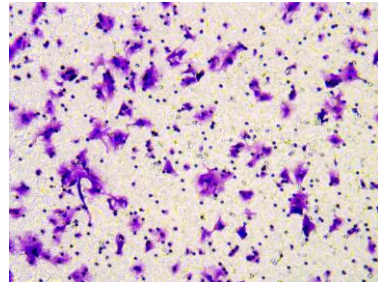

Invasion

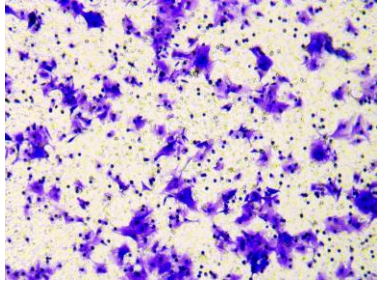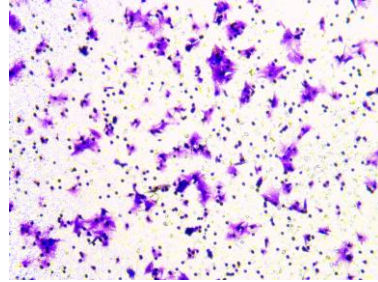

Supplement: Supplementary file 2 — Additional file 2: Figure S2. p-STAT3 inhibitor (S3I-201) inhibit the motility of HOX8B-overexpressing cells. with S3I-201 treated for 12 hours, the cell number of DLD1-HOXB8 was decreased both in migration and invasion assays, compared to control. [file 12935_2018_717_MOESM2_ESM.pdf]
